# Histone deacetylase inhibitors as novel anticancer therapeutics

**DOI:** 10.3747/co.v15i5.371

**Published:** 2008-10

**Authors:** D.R. Walkinshaw, X.J. Yang

**Affiliations:** * Rosalind and Morris Goodman Cancer Centre (formerly the McGill Cancer Centre, renamed in September 2008), McGill University, and Department of Medicine, McGill University Health Centre, Montreal, QC

**Keywords:** Histone deacetylase, tubulin acetylation, Hsp90, vorinostat, trichostatin A, tubacin, chromatin, epigenetics

## Abstract

Histone deacetylase inhibitors represent a promising new class of compounds for the treatment of cancer. Inhibitors of this kind currently under clinical evaluation mainly target the classical (Rpd3/Hda1) family of histone deacetylases. Of particular note, the U.S. Food and Drug Administration recently approved the first histone deacetylase inhibitor (Zolinza: Merck and Co., Whitehouse Station, NJ, U.S.A.) for the treatment of cutaneous T-cell lymphoma. Dozens of such inhibitors are now in phase ii–iii clinical trials, sometimes in combination with other chemotherapy drugs, for diverse cancer types, including both hematologic and solid tumours. In this mini-review, we provide an overview of the histone deacetylase superfamily, highlight the positive results of deacetylase inhibitors in cancer clinical trials, and comment on the prospects for the next generation of such inhibitors.

## 1. INTRODUCTION

**FIGURE 1:**
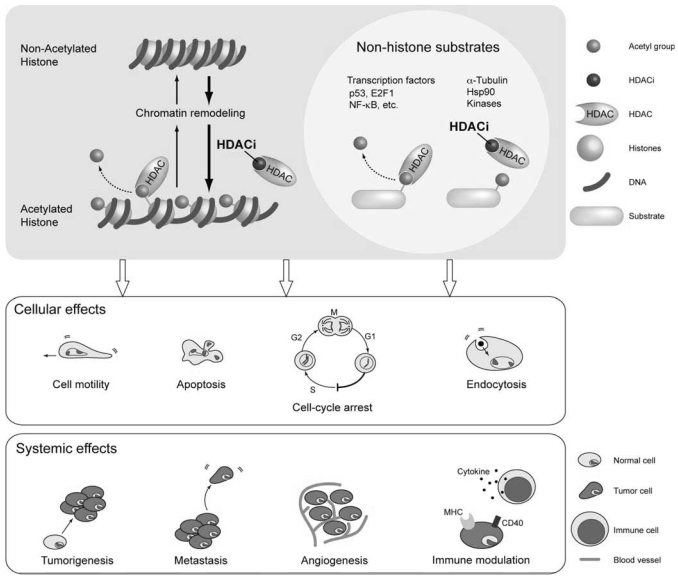
Schematic of the molecular and physiologic effects of treatment with histone deacetylase (HDAC) enzyme inhibitors (hdacis). Top panel: hdacis prevent the deacetylation of histones by hdacs (left), resulting in an open chromatin structure associated with transcriptional activation or in non-histone substrates (right), which increases acetylation of these hdac targets, resulting in changes in protein activity. Middle panel: Cellular effects of hdaci treatment. Lower panel: Systemic effects of hdaci treatment. hdaci = inhibitor of histone deacetylase; nf-κb = nuclear factor kappa B; hdac = histone deacetylase enzymes. Note that although we here highlight the “good side” of hdac inhibitors, inhibition of hdac activity may be detrimental in certain situations and thus give rise to side effects (the “dark side”). And in some cases, activation of hdacs may even be beneficial. (This figure was kindly produced by Dr. Noriko Uetani at the Rosalind and Morris Goodman Cancer Centre, McGill University, Montreal, QC. It was inspired by a previous model proposed by others 10.)

Aberrations in gene expression patterns lie at the heart of tumorigenesis. Specifically, inappropriate activation of oncogenes or silencing of tumour suppressor genes, or both, drive cellular proliferation and prevent apoptosis 1. Not surprisingly, many proteins involved in regulating transcription are themselves deregulated in cancer at the level of expression or activity. One such family of proteins is the histone deacetylase (hdac) enzymes. As the name implies, these enzymes remove acetyl groups from the side chain of specific lysine residues located mainly at the N-terminal flexible tails of core histones, thereby increasing the affinity of histone octamers for the dna that is wrapped around them (Figure 1). In addition, deacetylation affects internucleosomal histone–histone interactions and association of other proteins with chromatin. Because histone deacetylation results in a tightened chromatin structure, histone deacetylation is generally associated with transcriptional repression 2. Conversely, acetylation of histones by histone acetyltransferases relaxes chromatin (or alters its configuration) and leads to activation of transcription. In this way, hdacs can influence the epigenetic status of a cell, thereby changing gene expression patterns in the absence of mutations to the genome itself.

In addition to histones, hdacs target dozens of non-histone proteins, including many transcription factors (for example, the tumour suppressor p53), α-tubulin, heat shock protein 90 (Hsp90: an important anticancer drug target), and various signalling proteins (Figure 1) 3–5. Therefore, hdacs not only modulate the structure and dynamics of chromatin, but also play a key role in regulating other cellular processes such as cell motility, endocytosis, and membrane receptor signalling. In this regard, a more appropriate name would be lysine deacetylases, but for historical reasons, the term hdac has been (and is still widely) used even for enzymes that deacetylate non-histone proteins. Herein, we therefore use the term hdac to refer to lysine deacetylase enzymes exclusively.

The following five lines of evidence (among others) suggest that altered hdac activity may play a causative role in both tumorigenesis and metastasis (Figure 1) 6:

- First, histone hypoacetylation is characteristic of most cancers 7.
- Second, in leukemia, hdacs are often aberrantly recruited to gene promoters by fusion proteins derived from chromosomal translocations 8,9.
- Third, overexpression of specific hdacs occurs in several cancer types, and the growth of cell lines derived from these tumours is suppressed by small interfering rna (sirna)–mediated knockdown of the overexpressed hdac 10.
- Fourth, elevated expression of hdacs may block the function of tumour suppressor proteins such as p53 11.
- Fifth and finally, in addition to affecting chromatin structure and regulating gene expression and genomic stability, hdacs target oncogenic regulators such as Hsp90 that are important for cancer development 12,13.

With the benefit of hindsight, it is not surprising that early studies on hdac inhibitors (hdacis) revealed their selective toxicity toward cancer cells, a property that positioned them as promising anticancer therapeutics. In the past decade or so, various hdacis have entered the clinical trial stage for a variety of cancers 14. In this mini-review, we provide an overview of the human hdac superfamily, describe recent success in clinical trials for hdacis, and discuss the potential of class- and isotype-selective hdaci.

## 2. THE HDAC SUPERFAMILY

The 18 known human hdacs are grouped into two families and four classes that differ from each other in terms of structure and biologic activity 15,16. The Rpd3/Hda1 (“classical”) family is composed of class i (hdac1, hdac2, hdac3, and hdac8), class iia (hdac4, hdac5, hdac7, and hdac9), class iib (hdac 6 and hdac10), and class iv (hdac11). The class iii hdacs (sirt1 through sirt7) comprise the sirtuin (Sir2-related protein) family (Table I). The family names were derived from the yeast deacetylase prototypes Rpd3 (reduced potassium dependency 3), Hda1 (histone deacetylase 1), and Sir2 (silent information regulator 2). Within each family, members display sequence similarity in the deacetylase domain, but the two families show no homology at all. In addition, the two families have entirely different catalytic mechanisms and are thus not targeted by the same inhibitor molecules 17.

The term hdaci is often used to describe compounds that inhibit the classical hdacs of classes i, ii, and iv—compounds to which sirtuins are insensitive (Table I). Owing to their much more advanced clinical progress, we focus primarily on inhibitors of Rpd3/Hda1—the “classical” hdacs. Compounds targeting sirtuins are briefly discussed toward the end of this paper.

Class i hdacs (excluding hdac8) are subunits of multi-protein chromatin-modifying complexes 18. Class iia hdacs shuttle between the nucleus and the cytoplasm in response to extracellular signals; they are unique among hdacs for apparently weak deacetylase activity of vertebrate homologs 19–21. The class iib member, hdac6, is found mostly in the cytoplasm, where it deacetylates cytoskeletal proteins such as α-tubulin and cortactin 22,23. In sum, the Rpd3/Hda1 hdacs play roles in many cellular processes, including proliferation, differentiation, apoptosis, autophagy, cell motility, and stress responses 16.

## 3. HDAC INHIBITORS AS ANTICANCER DRUGS

The hdacis represent a broad family of chemical compounds that comprises four main structural classes:

- Short-chain fatty acids [butyrate and valproic acid (vpa)]
- Hydroxamates [trichostatin A, saha (sub-eroylanilide hydroxamic acid or vorinostat), LBH589 (panobinostat), PXD101 (belinostat), oxamflatin, and tubacin]
- Benzamides [SNDX275 (MS275) and MGCD0103]
- Cyclic tetrapeptides [FK228 (romidepsin), trapoxin A, and apicidin]

Not only do these compounds vary in structure, they also have distinct affinities for various hdacs. Hydroxamates are the only known pan-hdacis, with the ability to target all classical hdacs. In contrast, butyrate, vpa, and trapoxin A target class i and iia hdacs. Other compounds are more specific: SNDX275 inhibits class i hdacs (but not hdac8), romidepsin preferentially targets hdac1 and hdac2, and tubacin selectively inhibits hdac6 10. The chemical structures and pharmacokinetic properties of hdacis have been extensively reviewed elsewhere 24.

In the late 1970s and then in the early 1990s, a small number of studies ushered in the era of hdacis as chemotherapeutic agents. The hdac inhibitory activity of the naturally occurring compounds butyrate and trichostatin A (tsa) was found to be responsible for their ability to cause differentiation and cell cycle arrest of transformed cells 25,26. Later, chemically synthesized hydroxamates (including saha), which were known to induce differentiation or apoptosis of cancer cell lines (or both), were also identified as hdacis 27. These cell culture results have been recapitulated in animal models of cancer and in clinical trials with human cancer patients. Of note, saha was approved by the U.S. Food and Drug Administration in October 2006 under the trade name Zolinza (Merck and Co., Whitehouse Station, NJ, U.S.A.), for the treatment of cutaneous T-cell lymphoma 28. Zolinza and other hdacis are achieving successful results in phase ii and iii clinical trials for a wide range of cancers 9,10,14,29, including chronic myelogenous leukemia [panobinostat (Faridak: Novartis Pharmaceuticals, St. Louis, MO, U.S.A.)], acute myeloid leukemia (MGCD0103: MethylGene, Montreal, QC; belinostat: TopoTarget U.K., Abingdon, U.K.; SNDX275: Bayer Schering Pharma AG, Berlin, Germany), cutaneous T-cell lymphoma (panobinostat; romidepsin: Astellas Pharma, Tokyo, Japan), B-cell lymphoma (Zolinza, belinostat), breast cancer (Zolinza), prostate cancer (Zolinza, romidepsin), multiple myeloma (Zolinza, panobinostat, romidepsin), pancreatic cancer (MGCD0103, romidepsin), colorectal cancer (Zolinza), thyroid cancer (belinostat, romidepsin), non-small-cell lung cancer [Zolinza; AN9 (a butyrate derivative): Bar-Ilan Research and Development Co., Ramat-Gan, Israel], and both Hodgkin and non-Hodgkin lymphoma (Zolinza, panobinostat, belinostat, MGCD0103).

### 3.1 Mechanism of HDAC Inhibitor-Mediated Tumour Suppression

Because of the function of hdacs as canonical transcriptional co-repressors, the global transcription changes elicited by hdacis are somewhat surprising. It is estimated that the expression level of approximately 2%–10% of genes is altered by hdaci treatment, with as many genes downregulated as upregulated 30. The most studied of the upregulated genes is *CDKN1A,* which encodes the cell cycle inhibitor kinase p21. Although the re-expression of this gene explains the antiproliferative effect of hdaci, cell-cycle arrest is not crucial for hdaci-mediated tumour suppression because hdacis cause cell-cycle arrest in normal and cancer cells alike, and unlike many chemotherapeutic drugs, hdacis can kill proliferating cancer cells^30^.

As for the method of this tumour cell killing, there are a plethora of routes through which it can occur. The main process through which hdacis induce cancer cell cytotoxicity is apoptosis (programmed cell death). Apoptosis can occur through the extrinsic (death-receptor) or the intrinsic (characterized by cytochrome C release from mitochondria) pathway 30. Interestingly, the potent pro-apoptotic transcription factor p53 does not appear to be required for hdaci-induced apoptosis. Instead, the pro-apoptotic Bax and BH3-only proteins Bid or Bim are necessary for this effect 31,32. Moreover, overexpression of the anti- apoptotic proteins Bcl2 or BclX_L_ abrogates apoptosis induction by hdaci 31.

Apoptosis induced by hdaci also depends on elevations in reactive oxygen species (ros) caused by hdaci treatment. This phenomenon likely reflects the inability of tumour cells to upregulate thioredoxin, an antioxidant protein, during hdaci-induced ros accumulation 33.

The hdacis can also induce genomic instability by impairing dna damage repair 34 and by causing mitotic catastrophe (mis-segregation of chromosomes because of defects in the mitotic spindle checkpoint). Further, in experimental conditions of blocked apoptosis, hdacis can still induce cancer cell death by an autophagy-dependent process 35. This latter finding is exciting, because it indicates that hdacis may still be effective against cancers with apoptosis defects 30.

The antitumorigenic properties of hdacis also extend to more systemic effects involving the tumour blood supply, metastatic potential, and host immune response. The hdacis inhibit angiogenesis by repressing the expression of pro-angiogenic factors such as vascular endothelial growth factor and by reducing the sensitivity of endothelial cells for angiogenic stimuli 10,36. In addition to preventing the new blood supply crucial to a growing tumour, hdacis also inhibit the expression of matrix metalloproteinase enzymes, which degrade the basement membrane surrounding tumours, an important initial step in metastasis 10. Moreover, stimulation of the host immune response by hdacis can sensitize tumour cells for destruction by the immune system 37. Therefore, hdacis can induce diverse cellular and physiologic processes that act in concert to prevent the initiation and progression of cancer.

### 3.2 Potential of Isotype- or Class-Selective HDAC Inhibitors

The fact that class i-selective hdacis (for example, MGCD0103, SNDX275, and romidepsin) possess antitumorigenic capacities strongly suggests that inhibition of class i hdacs may be sufficient for chemotherapy. In support of this hypothesis, genetic deletion and sirna-mediated knockdown studies support the non-redundant role of these hdacs in controlling cell proliferation and survival 6. In particular, *HDAC1* knockout mice die during embryogenesis because of a lack of cell proliferation 38; gene trap–mediated disruption of *HDAC2* reduces tumour growth in a tumour-prone mouse model 39; and loss of *HDAC3* causes embryonic lethality associated with decreased proliferation, dna damage, and apoptosis 34. Moreover, overexpression of each of these hdacs is seen in several cancer types 6.

In addition to these class i hdacs, the class iib member hdac6 is likely also an important target of hdaci-mediated cancer suppression because of its ability to control cell migration through deacetylation of α-tubulin and cortactin, and through its ability to deacetylate Hsp90 and to disrupt that protein’s function as a chaperone for oncogene products 6.

In contrast, there is much less evidence implicating class iia hdacs in tumorigenesis. In fact, inhibition of class iia hdacs (as occurs during treatment with pan-hdacis such as vorinostat, tsa, vpa, or butyrate) could lead to a host of unwanted side effects, as inferred from the role of class iia hdacs in limiting the activation of myocyte enhancer factor 2 (*Mef2*) target genes. Genetic deletion of each class iia member causes unrestrained *MEF2* activation, leading to skeletal defects, pathologic cardiac hypertrophy, or collapse of the vascular system 40. Thus, it will be of interest to compare long-term recipients of class i-selective hdacis with recipients of pan-hdacis to see if patients in the latter group selectively suffer from these types of side effects.

Although it has not yet been established whether class i–selective hdacis elicit fewer side effects than pan-hdacis do, it is clear that hdacis in general are associated with certain toxicities, including fatigue, nausea, diarrhea, and vomiting 9,41. The issue of hdaci toxicity must be addressed if drugs with a broad range of clinical utilities in diverse patients are to be developed. Currently, only one truly specific hdaci has been developed. Tubacin selectively targets hdac6, and it can induce apoptosis of multiple myeloma cells *in vitro* 42 and inhibit transforming growth factor β–induced epithelial-to-mesenchymal transition 43. However, this compound has yet to enter clinical trials, and thus its toxicity profile is not yet known. Of course, whether isotype-selective hdacis will reduce toxic side effects will remain unknown until such agents are developed and tested. Yet, even if the next generation of hdacis lack better toxicity profiles, they will nevertheless be instrumental in elucidating the function or functions of individual hdacs involved in various types and stages of cancer and in other human diseases.

The specific hdaci used for specific cases may also be of critical importance independent of the hdacs it inhibits. A recent study demonstrated that specific hdacis can have dramatically different effects depending on the molecular profile of the tumour. In a mouse model of B cell lymphoma, Bcl2 overexpression blocks the ability of the hydroxamates, but not of the cyclic tetrapeptide romidepsin to prevent lymphoma 44. Whether this differential blocking is the result of different substrate specificities is unknown, but it at least points to the phenomenon that not all hdacis are equal in terms of biologic activity. This result also provides a glimpse into a future, when therapies are tailored to the individual gene expression profiles of tumours in patients 45. In this case, a lymphoma patient with overexpressed Bcl2 would be better served with romidepsin than with a hydroxamate hdaci.

### 3.3 Use of HDAC Inhibitor in Combination with Other Chemotherapies

The optimal use of hdacis is highly likely to be in combination with other anticancer therapies. Several studies have shown that hdacis can synergize with a large set of other chemotherapeutic drugs such as cisplatin, etoposide, bortezomib, and gemcitabine. Other therapeutics that exhibit an additive or synergistic effect when used with hdacis include differentiation agents such as retinoic acid (ra) and all-trans retinoic acid (atra) for the treatment of leukemic cells; imatinib, an inhibitor of the Bcr-Abl tyrosine kinase expressed by the Philadelphia chromosome in chronic myelogenous leukemia; the breast cancer drug trastuzumab, a monoclonal antibody against the her2/*neu* (ErbB2) receptor; and 17AAG, an inhibitor of the oncogenic protein chaperone Hsp90 10,30. In all these cases, at least *in vitro,* these drugs cooperate with hdacis to enhance apoptosis—or, in the case of ra and atra, differentiation—of cancer cells. Further, hdacis can induce differentiation or apoptosis, respectively, of ra- or imatinib-resistant cells. In addition, hdacis enhance the radiosensitivity of gamma- or ultraviolet-irradiated tumours and can bolster the effect of the trail (tumour necrosis factor–related apoptosis-inducing ligand) death receptor ligand on tumour cell death 10,30. A link is emerging between hdac activity and dna methylation in controlling gene expression 18; it is therefore not surprising that the combination of hdacis and dna methyltransferase inhibitors also exhibit enhanced anti-neoplastic activity 10,30.

Excitingly, it appears that the principle of synergism between hdacis and other compounds can be recapitulated in a single hybrid compound. The first compound of this kind, termed triciferol, in which the side chain of 1,25-vitamin D_3_ (the active form) is replaced with the dienyl hydroxamic acid of tsa 46, possesses characteristics of both 1,25-D_3_ (vitamin D receptor agonism) and tsa (histone hyperacetylation) and exhibits effects similar to co-treatment with the two original compounds in terms of cancer cell cytotoxicity *in vitro* 46,47. It will be greatly interesting to see if this compound is effective in animal studies and human clinical trials, and also to see whether hdacis can be combined with other drugs to form new antitumorigenic hybrid compounds.

## 4. SUMMARY AND FUTURE PERSPECTIVES

The flurry of activity surrounding the development of hdacis has paralleled the rapid expansion over the past decade of research into the molecular details of hdac activity and the biologic roles of these enzymes. The discovery of their importance to multiple stages of tumour initiation and progression provided a strong rationale for the use of compounds targeting these epigenetic regulators in cancer treatment. With current compounds exhibiting positive clinical results, and one hdaci already on the market in the United States, it is clear that hdacis will likely represent an important class of chemotherapeutic drugs in the near future. Whether newer hdacis, possibly isotype-selective versions, will ultimately supplant the current collection remains to be seen, but would not be surprising given the modest toxicity problems associated with today’s generation of hdacis. Nevertheless, the clinical utility of altering hdac activity has been unambiguously demonstrated.

Although we have focused on inhibitors of the Rpd3/Hda1 family of deacetylases herein, modulators of sirtuin activity (Table I) may prove to be important therapeutics for the treatment of various diseases, including cancer 48. For instance, the sirt1 activator resveratrol—a polyphenol famously contained in red wine—can prevent cancer by targeting multiple stages of progression in mice 49. In addition, it was recently reported that tenovins, novel sirt1 and sirt2 inhibitors, increase apoptosis of cancer cell lines and delay tumour growth in mice, in part by increasing the acetylation and transcriptional activity of *TP53* 50. Despite these advances, sirtuin-targeting compounds (inhibitors and activators alike) lag behind conventional hdacis in terms of their progression from the lab to the pharmacy. Their continued development, alongside that of hdacis, is crucial to fully exploit the importance of protein acetylation in cancer.

The present mini-review has dealt primarily with the idea that increasing acetylation of histones or other proteins through hdac inhibition is always desirable, but it is important to note that it is the balance of hdac and histone acetyltransferase activities, and hence the relative acetylation levels of key proteins (which may vary in different tissues and disease states) that is of ultimate importance. For this reason, cases may occur in which too much acetylation is pathologic, and inhibitors of histone acetyltransferases may therefore be efficacious 51,52. Indeed, inhibition of the acetyltransferase activity of p300 by curcumin—the polyphenol contained in the curry spice turmeric—blocks cardiac hypertrophy and heart failure in mouse models 53. Curcumin has also been touted as a potential anticancer compound, although it is not yet known whether its potential activity is a function of its histone acetyltransferase inhibitory function 54.

Although cancer has traditionally been viewed as a genetic disease, the perspective of cancer as an epigenetic disease is rapidly emerging. Thus, in addition to mutations in the dna code, it is increasingly clear that cancer can result from aberrations in chromatin and chromatin-modifying proteins such as hdacs. As a result, hdacis represent one promising subgroup of compounds for epigenetic therapy. The elucidation of the mechanisms through which hdacis suppress tumours, the discovery of the particular hdacs that are involved in each stage or type of cancer, the continued development of isotype-selective hdacis, and the optimization of hdacis as a component of combination chemotherapy represent important breakthroughs that will undoubtedly occur in the near future. With continued research and development, hdacis are likely to figure prominently in cancer treatment plans in the near future.

## Figures and Tables

**TABLE I:** Comparison of the two families of histone deacetylase (HDAC) enzymes

Member Family	hdac1–hdac11 Rpd3/Hda1 (“classical”)	Sirtuin (sirt1–sirt7) Sir2 (sirtuin)
Class	i, ii, and iv	iii
Cofactor	Zn^2+^	nad^+^
Activator	?	Resveratrol
Inhibitor	Butyrate, tsa, and saha	Sirtinol, EX-527, and tenovin
Inhibitor for clinical trial	Yes	Yes
U.S. fda approval	saha	Not yet

nad = nicotinamide adenine dinucleotide; tsa = trichostatin A; saha = suberoylanilide hydroxamic acid; U.S. fda = U.S. Food and Drug Administration.
